# Sperm traits on *in vitro* production (IVP) of bovine embryos: Too much of anything is good for nothing

**DOI:** 10.1371/journal.pone.0200273

**Published:** 2018-07-10

**Authors:** Adriano Felipe Perez Siqueira, Letícia Signori de Castro, Patrícia Monken de Assis, Luana de Cássia Bicudo, Camilla Mota Mendes, Marcílio Nichi, José Antônio Visintin, Mayra Elena Ortiz D’Ávila Assumpção

**Affiliations:** 1 Laboratory of Spermatozoa Biology, Department of Animal Reproduction, School of Veterinary Medicine and Animal Science, University of Sao Paulo, Sao Paulo, Brazil; 2 Laboratory of *in vitro* Fertilization, Cloning and Animal Transgenesis, Department of Animal Reproduction, School of Veterinary Medicine and Animal Science, University of Sao Paulo, Sao Paulo, Brazil; 3 Laboratory of Andrology, Department of Animal Reproduction, School of Veterinary Medicine and Animal Science, University of Sao Paulo, Sao Paulo, Brazil; University of Florida, UNITED STATES

## Abstract

Sperm samples used on fertilization strongly influence the *in vitro* production (IVP) rates. However, sperm traits behind this effect are not stated consistently until now. This study aimed to evaluate the isolated and combined effect of some sperm traits (MB: total motility before Percoll^®^ gradient, MA: total motility after Percoll^®^ gradient, AI: acrosome integrity, MI: membrane integrity, MP: mitochondrial membrane potential, and CR: chromatin resistance) on IVP rates. This is the first study focusing on the isolated effect of distinct traits. For this purpose, the experiment was divided in three steps. In first step, to study behavior of traits sperm samples (n = 63 batches) were analyzed and ranked based on each trait. In second step, samples ranked were selected from target ranks regions and allocated in groups of four to five batches, creating Higher and Lower groups, according to two different approaches. One aimed to form groups that differed to all sperm traits simultaneously (effect of combined traits). The other aimed to form groups that differed only to a single sperm trait while no differences were observed for the remaining traits (effect of each isolated trait). In third step, for each group successfully formed in step 2, sperm samples were individually and prospectively used for IVP. Cleavage, embryo development and blastocyst rates were recorded and compared between Higher and Lower of respective trait groups. Surprisingly, evaluation of isolated effects revealed that lower levels of MB, AI and MP resulted in higher embryo development and blastocyst rates (p<0.05), which was not observed on cleavage rate. We conclude that sperm traits strongly influence embryo development after *in vitro* fertilization (IVF), affecting the zygote competence to achieve blastocyst stage. Individually, levels of MB, AI or MP could be some of the key traits that may define IVP efficiency on current systems of embryo production.

## Introduction

*In vitro* production (IVP) of bovine embryos has allowed the use of a waste biological resource from valuable females to increase their offspring in breeding programs, particularly in Brazil [1,2]. Nevertheless, more than half of IVP embryos fail to reach the blastocyst stage during *in vitro* culture (IVC). Intuitively and mistakenly, this suggests that IVC step is the main responsible for the IVP failures [1]. However, Rizos et al. [3] showed that during IVC, *in vivo* matured oocytes achieved higher blastocyst rate than *in vitro* oocytes (58.2% *versus* 38,9%) and *in vivo* fertilized oocytes result in higher blastocyst rate compared to *in vitro* fertilized ones (73.9% *versus* 58.2%), regardless the similar cleavage rates. Such results may indicate that problems during *in vitro* maturation (IVM) and *in vitro* fertilization (IVF) steps could potentially impair the blastocyst production mainly during embryo development rather than first cleavages.

In addition to IVF shortcomings, the bull effect has been generally related as a cause of variation on IVP rates, including differences between batches from same bull [4–18]. In this context, attempts to select sperm samples with improved *in vitro* performance based on sperm features would be an interesting tool to indicate, beforehand, IVP performance and improve embryo production yields [9]. However, to predict the fertilization ability of a given sperm sample by sperm traits analysis is still a long way to achieve any substantial results using the current approaches. This is probably due to the unknown importance of sperm traits analyzed and the disregards of possible confounding effects such as bull effect and interactions among traits.

In attempt to better understand the relation between sperm traits and IVP rates, we chose sperm traits previously suggested as possible predictor candidates for IVP performance: motility [9,14,16,17,19], status of acrosome [7–9,13,14,16,19], plasma membrane [9,13,14,16,19], chromatin [16,19–21] and mitochondria [9,21], and we evaluated their effect on IVP. These sperm traits play fundamental roles during fertilization process and embryo development and together imply the status of the main function and cellular structures of spermatozoa.

The present study was designed to identify which sperm traits are important to determinate IVP yields. With this information, it will be possible to select samples that could provide higher IVP yield to guide the improvement of sperm production and handling used to IVP, and also to elaborate strategies to upgrade IVF step. While these traits have been previously studied, some confounding effects were disregarded. To the best of our knowledge, this is the first report in which the effect of bull has been controlled and the interactions among distinct traits have been studied on IVP.

## Materials and methods

This research was approved by Ethic Committee in the use of animals of the School of Veterinary Medicine and Animal Science of University of São Paulo, protocol number: 2720/2012

### Experimental design

**Fig 1:**
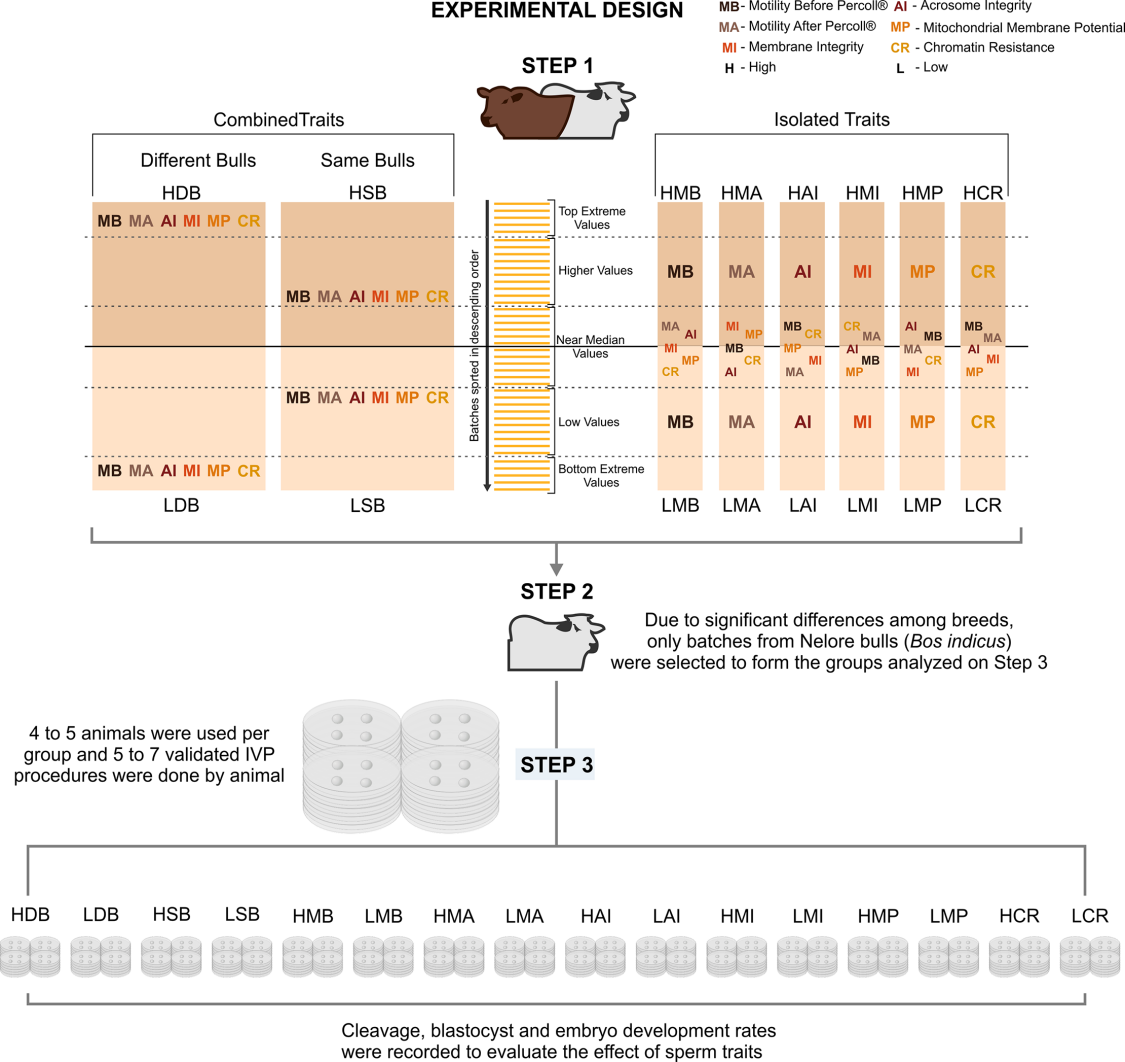
Experimental design, batches rank, representative areas used in batches selection for each step. HMB, Higher Motility Before Percoll^®^; LMB, Lower Motility Before Percoll^®^; HMA, Higher Motility After Percoll^®^; LMA, Lower Motility After Percoll^®^; HMI, Higher Membrane Integrity; LMI, Lower Membrane Integrity; HAI, Higher Acrosome Integrity; LAI, Lower Acrosome Integrity; HMP, Higher Mitochondrial Membrane Potential; LMP, Lower Mitochondrial Membrane Potential; HCR, Higher Chromatin Resistance; LCV, Lower Chromatin Resistance; HSB, Higher Sperm Traits Profile among same bulls; LSB, Lower Sperm Traits Profile among same bulls; HDB, Higher Sperm Traits Profile among different bulls; LDB, Lower Sperm Traits Profile among different bulls.

The sperm traits selected for this study were total motility before Percoll^®^ Gradient (MB), total motility after Percoll^®^ Gradient (MA), acrosome integrity (AI), membrane integrity (MI), mitochondrial membrane potential (MP), and chromatin resistance (CR). Only total motility was evaluated before and after Percoll^®^ Gradient selection, the other sperm traits were evaluated just after Percoll^®^ selection. To evaluate their effects on IVP performance, the experiment was divided in three steps (Fig 1). In the first step, sperm samples analyzed were ranked for each trait to study the behavior and variation of traits in samples and the semen database was built. In the second step, samples were ranked for each trait, selected from target regions of the ranks and allocated in groups, according to two different approaches. One approach aimed to form groups that differed to all sperm traits simultaneously (effect of combined traits, with extreme values in all ranks). The other approach aimed to form groups that differed only to a single sperm trait while no differences were observed for the remaining traits (effect of each isolated trait, higher and lower median values of ranks). In the third step, for each group formed of step 2, sperm samples were individually and prospectively used for IVP.

#### Step 1: Building the semen database

To study the effect of sperm traits on IVP yields was necessary, first, build a semen database to analyze the variation of each trait in the samples after Percoll^®^ Gradient selection, and check whether the behavior between traits is dependent (correlations presence) or independent (correlations absence). For this purpose, 63 batches from commercial available semen straws from 35 bulls of different breeds, purchased or donated from Artificial Insemination Centers from Brazil, were evaluated by flow cytometry. Samples were analyzed in four replicates (four straws analyzed separately per sample; n = 252). The median of the four replicates was used to represent the value and the sperm traits profile of each batch. Then, batches were sorted in descending order for each sperm trait analyzed, resulting in six ranks (MB, MA, AI, MI, MP, CR) to evaluate variation on the levels of traits and distribution of breeds and bulls.

#### Step 2: Samples selection and grouping

The aim of Step 2 was to verify the possibility to group samples from the ranks according to two different approaches. 1) evaluate the effect of combined traits (i.e., groups that differ for all sperm traits simultaneously) using different bulls in each group and using the same bulls in both groups but with different batches; 2) evaluate the effect of isolated trait (i.e., groups that differ only for one sperm trait, with no differences for the other traits).

Due to significant differences among breeds on step 1 (data not shown), only batches from Nelore bulls (*Bos indicus*) were used on step 2. Nelore breed was selected because they were the majority of samples analyzed (48 batches from 21 bulls).

For the effect of combined traits, samples from extreme positions in the rank for all the six traits, top extreme values and bottom extreme values, were selected to form higher and lower groups (Fig 1). In this case, groups were formed with different bulls in each group. To eliminate the bull effect on IVP (Step 3) we also formed groups with batches from the same bulls with extreme values (Top Extreme Batch and Bottom Extreme Batch). The effect was considered combined when the higher and the lower group differed significantly (p<0.05) for the values of all traits analyzed.

For the effect of isolated traits, samples were selected among those with values that were higher (Higher isolated effect group) or lower (Lower isolated effect group) than the median for this trait, but similar for the remaining traits, (closer to the median–Fig 1). In this case, a selected trait was considered isolated when the values of higher and the lower group differed significantly (p<0.05) and did not differ (p>0.10) for the values of the remaining traits. Only one batch of each bull could be used in a same group.

To prevent that a confounding bull effect was assumed as a sperm trait effect, at least four samples of different bulls were selected to form each group (combined and isolated groups). The acronyms of higher and lower groups of each effect studied were presented on Table 1.

**Table 1:** Acronyms of higher and lower groups composed to each effect studied.

Effect Studied	Higher Group	Lower Group
Isolated Effect of Motility Before Percoll^®^	HMB	LMB
Isolated Effect of Motility After Percoll^®^	HMA	LMA
Isolated Effect of Acrosome Integrity	HAI	LAI
Isolated Effect of Membrane Integrity	HMI	LMI
Isolated Effect of Mitochondrial Membrane Potential	HMP	LMP
Isolated Effect of Chromatin Resistance	HCR	LCR
Combined Effect among Same Bulls	HSB	LSB
Combined Effect among Different Bulls	HDB	LDB

#### Step 3: Sperm traits effect on IVP yield

The aim of Step 3 was to evaluate the effects of groups formed in step 2 on IVP yields. Five to seven procedures of IVP for each sample from higher and lower groups of effects described on Table 1 were carried out (n = 365). The cleavage, blastocyst and embryo development rates were recorded to evaluate the effect of sperm traits. One bull with known IVP performance was used as control for every IVP procedure. Only IVP procedures with blastocyst rates higher than 20% for this bull were considered in the study. To remove potential atypical IVP rates, the highest and the lowest blastocyst rate of each sample were excluded from analysis.

### Sperm preparation

Semen straws were thawed at 37ºC for 30 seconds. Motile sperm cells were selected by discontinuous Percoll^®^ density gradient (400μl Percoll^®^ 45% over 400μl Percoll^®^ 90%, warmed at 38.5ºC) for 6 minutes at 9000g at room temperature. Cells from the sediment were recovered (100 μL) and washed in Fert-TALP warmed at 38.5ºC [22] without capacitation inductors, for 3 minutes at 9000g at room temperature. Washed sperm cells were counted in Neubauer chamber and diluted in the appropriate volume of Fert-TALP without capacitation inductors to achieve a final concentration of 25 X 10^6^ motile spermatozoa/mL. This final diluted sample was evaluated for acrosome integrity, membrane integrity, mitochondrial membrane potential, and chromatin resistance to acid denaturation, in step 1 and the same procedure was applied in step 3 for IVF.

### Motility analysis

The percentage of total motile sperm cells, before (MB) and after (MA) Percoll^®^, was estimated placing 10 μl of semen sample on a warmed slide (38.5 ºC) and covered with a warmed cover slip. The slide was evaluated using a phase contrast microscopy at 100X magnification. The motility of all samples was estimated by a single evaluator.

### Acrosome integrity (AI), membrane integrity (MI), mitochondrial membrane potential (MP), and chromatin resistance (CR) analysis

Acrosome and membrane integrities were determined simultaneously in the same sample. This was performed by incubating 187,500 cells for 5 minutes with 10μM of propidium iodide (PI) associated with 5μg of fluroscein-conjugated psium sativum (FITC-PSA). PI emits red fluorescence for damaged plasma membrane, while FITC-PSA emits green fluorescence for damaged acrosome.

For mitochondrial membrane potential assessment, 187,500 prepared cells were incubated for 5 minutes with 1μM of tetraethylbenzimidazolycarbocyanine iodide (JC-1) [23]. This probe emits green fluorescence for low mitochondrial potential and red/orange fluorescence for high mitochondrial potential.

As control for acrosome and membrane integrity and high mitochondrial membrane potential (control 1), one sample with high percentage of AI, MI and MP was prepared as described above. As a control of damaged acrosome and membrane, and lack of mitochondrial membrane potential (control 2), part of the control 1 sample was submitted to freezing/thawing cycles (1’ in liquid nitrogen/ 1’ in water bath at 60ºC, 5 times).

Chromatin resistance analysis was performed according to previous work of our group [24]. Briefly, 375,000 of prepared cells were added to 50μL of TNE buffer (10mM Tris-HCl, 0.15M NaCl, 1mM EDTA disodium pH = 7.4) and 100μL of acid detergent solution (0.08M HCl, 0.15M NaCl, 0.1% (v/v) Triton X-100, pH = 1.4). After 30 seconds, 300μL of Acridine Orange solution (6μg/mL in 0.1M citric acid, 0.2M Na_2_HPO_4_, 1mM de EDTA, 0.15M NaCl, pH = 6.0) were added, and evaluation were performed by flow cytometry, 3–5 minutes following Acridine Orange Solution addition. As control of chromatin resistance (control 1), a sample was prepared as described above in sperm preparation and as control of chromatin damaged (control 2) part of control 1 was incubated with HCl (1.2M in acid detergent solution, pH = 0.1) for one minute.

All flow cytometry analyses were performed in a Guava EasyCyte^TM^ Mini System (Guava^®^ Technologies, Hayward CA, USA) 20mW 488nm argon excitation laser. Probes were purchased from Sigma and Molecular Probes.

Cytometry data were analyzed using the FlowJo software (version 10.2 Flow Cytometry Analysis Software–Tree Star INc., Ashland, Oregon, USA). Compensation parameters were held for all samples. A total of 20,000 events per sample were analyzed. Controls 1 and 2, of each analysis were mixed in different proportions (1:3; 1:1; 3:1). Controls and proportions were analyzed, cells were identified and selected excluding debris, probes particles and non-single sperm events applying a gate on forward scatter (FSC) *vs* green florescence dot plot (log mode) around the single sperm events to acrosome, membrane, and mitochondrial analysis. For chromatin analysis, the single sperm events were selected applying a gate on forward scatter (FSC) *vs* red florescence dot plot (log mode). Negative and positive thresholds and gates for each analysis were set up to achieve the highest determination coefficient on controls and proportions analysis. All traits analysis reached coefficients higher than 0.96. These set ups were applied for all samples.

### *In vitro* embryo production

Ovaries were collected in a slaughterhouse and transported to the laboratory 2–4 h after collection, in saline solution (0.9% at 30ºC). Follicles of 2–8 mm of diameter were aspirated using an 18-gauge needle and 5 mL syringe. After three washes in holding medium (TCM199 Hepes supplemented with 10% fetal calf serum (FCS, Gibco), 22μg/mL pyruvate and 50μg/mL gentamycin) and three washes in maturation medium (TCM199 Bicarbonate supplemented with 10% FCS, 22μg/mL pyruvate and 50μg/mL gentamycin, 0.5μg/mL FSH Folltropin V (Vetrepharm, Inc. Belleville, ON, Canada) and 50μg/mL HCG (Vetecor Laboratories, Calier, Spain) and 1μg/mL of 17β-estradiol), groups of 20 cumulus oocyte complexes (COCs) were placed in 90μL maturation medium droplets, mineral oil covered, during 22–24 hours at 38.5ºC, 5% CO_2_ in air, and high humidity.

After maturation, COCs were washed three times in pre-IVF medium (TCM199 Hepes supplemented with 0.003% of BSA-V, 22μg/mL pyruvate and 50μg/mL gentamycin) and three times Fert-TALP [22]. Groups of20 matured COCs were placed in 90μL Fert-TALP droplets, mineral oil covered, co-incubated with 5μL of prepared sperm (final concentration in IVF droplets = 1.25 X 10^6^ motile spermatozoa/mL) during 20 hours at 38.5ºC, 5% CO_2_ in air, and high humidity. Four droplets were inseminated with prepared sperm from each batch per IVP procedure (60–90 COCs/sample/IVP procedure). Samples were randomly divided in each IVP procedure.

After IVF, presumptive zygotes were gentle denuded by repeated pipetting in pre-IVF medium, washed three times in pre-IVF medium and three times in KSOM medium (Millipore Corporation, New Bedford, MA, USA), grouped in 20 presumptive zygotes and placed in 60μL KSOM droplets, under mineral oil, cultured during 8 days at 38.5ºC, 5% CO_2_, 5% O_2_, 90% N_2_, and high humidity. On third day of IVC (D3), 30μL of KSOM medium were remove from each droplet and replaced by 30μl of KSOM medium supplemented with 10% FCS (5% of FCS final concentration), and on fifth day, 30μL of KSOM medium supplemented with 5% FCS were added in each droplet. Cleavage rate (number of cleaved embryos/number of oocytes inseminated) was assessed on D3, and blastocyst rate (number of embryos that achieved early blastocyst or more advanced stage/number of oocytes inseminated) was recorded on D8. Embryo development rate was defined as the number of embryos that achieved early blastocyst or more advanced stage/number of cleaved embryos.

### Statistical analysis

The behavior between sperm traits in the step 1 was tested using Spearman correlation analysis. Differences between groups in the step 2, as well as data of the cleavage, blastocyst and embryo development rates from step 3 were compared using de Mann-Whitney-Wilcoxon test. A p value lower than 0.05 was considered significant. In the step 2 a p value higher than 0.10 was considered to exclude a significant effect of other sperm traits on isolated effect study. Statistical analysis was performed using the software Statistical Analysis System 9.3 (SAS Institute, Cary, NC, USA).

## Results and discussion

### Step 1: Building the semen database

We found positive correlations among all sperm traits analyzed (Table 2). Associations among sperm traits agree with several previous studies [25–30]. Sperm traits analyzed showed a dependent behavior between them. Accordingly, the use of distinct methods to select motile spermatozoa, indirectly selects cells with intact acrosome and plasma membrane, higher mitochondrial membrane potential and undamaged chromatin [9,10,16,31]. Although these improvements, batches, bulls, and breeds showed differences on sperm traits levels after selection. Furthermore, a low coefficient of variation was observed among replicates from the same sample (Table 3), indicating that distinct straws from a same batch provided stable levels of sperm traits after Percoll^®^ gradient selection. This indicates high accuracy of flow cytometry analysis and high repeatability in the present study.

**Table 2:** Correlations coefficient (Rho) among percentages of sperm motility before Percoll^®^, motility after Percoll^®^, acrosome integrity, membrane integrity, mitochondrial membrane potential and chromatin resistance (Step 1; n = 252).

	MA	MI	AI	MP	CR
**MB**	0.28138 p < .0001	0.22833 p = 0.0004	0.19400 p = 0.0026	0.19024 p = 0.0033	0.22436 p = 0.0005
**MA**	0.26138 p < .0001	0.31109 p < .0001	0.26047 p < .0001	0.26630 p < .0001
**MI**			0.77817 p < .0001	0.75837 p < .0001	0.53284 p < .0001
**AI**				0.65305 p < .0001	0.50243 p < .0001
**MP**					0.45479 p < .0001

MB, total motility before Percoll^®^; MA, total motility after Percoll^®^; MI, membrane integrity; AI, acrosome integrity; MP, mitochondrial membrane potential; CR, chromatin resistance.

**Table 3:** Mean of coefficient of variation among four replicates of 63 batches.

MB	MA	MI	AI	MP	CR
23,01%	15,43%	7,35%	4,22%	8,26%	1,74%

MB, total motility before Percoll^®^; MA, total motility after Percoll^®^; AI, acrosome integrity; MI, membrane integrity; MP, mitochondrial membrane potential; CR, chromatin resistance.

### Step 2: Samples selection and grouping

Due to differences between breed (data not shown), in Step 2 only Nelore samples were selected. This strategy was adopted since breeds have a great effect on sperm quality [32]. To evaluate the effect of combined traits, samples selected either from the same bull with different batches or different bulls, differed simultaneously to all sperm traits analyzed (Table 4). However, it has been postulated that to predict bull fertility, traits selection should include repeatability measures and minimal correlation among them [33]. Regardless of the correlations between all sperm traits in our study, it was possible to isolate de effect of all traits analyzed, forming groups which differ only for values of the target trait (Table 4). The study of isolated effect on embryo production revealed unexpected impact of sperm traits on IVP. Therefore, this could be the first step to elucidate the complex relation between sperm traits and IVP yields.

**Table 4:** Comparison of sperm profile between groups. Comparison of motility before Percoll^®^ (MB), motility after Percoll^®^ (MA), acrosome integrity (AI), membrane integrity (MI), mitochondrial membrane potential (MP) and chromatin resistance (CR) between higher groups and lower groups of isolated and combined effects.

Effect Studied	SPERM TRAITS PROFILE COMPARISION					
	MB	MA	AI	MI	MP	CR
Isolated Effect of Motility Before Percoll^®^ (HMB X LMB)	●	○	○	○	○	○
Isolated Effect of Motility After Percoll^®^ (HMA X LMA)	○	●	○	○	○	○
Isolated Effect of Acrosome Integrity (HAI X LAI)	○	○	●	○	○	○
Isolated Effect of Membrane Integrity (HMI X LMI)	○	○	○	●	○	○
Isolated Effect of Mitochondrial Potential (HMP X LMP)	○	○	○	○	●	○
Isolated Effect of Chromatin Resistance (HCR X LCR)	○	○	○	○	○	●
Combined Effect among Same Bulls (HSB X LSB)	●	●	●	●	●	●
Combined Effect among Different Bulls (HDB X LDB)	●	●	●	●	●	●

●, significant difference between higher and lower groups (p<0.05); ○, absence of significant difference between higher and lower groups (p≥0.10); HMB, Higher Motility Before Percoll^®^; LMB, Lower Motility Before Percoll^®^; HMA, Higher Motility After Percoll^®^; LMA, Lower Motility After Percoll^®^; HMI, Higher Membrane Integrity; LMI, Lower Membrane Integrity; HAI, Higher Acrosome Integrity; LAI, Lower Acrosome Integrity; HMP, Higher Mitochondrial Membrane Potential; LMP, Lower Mitochondrial Membrane Potential; HCR, Higher Chromatin Resistance; LCV, Lower Chromatin Resistance; HSB, Higher Sperm Traits Profile among same bulls; LSB, Lower Sperm Traits Profile among same bulls; HDB, Higher Sperm Traits Profile among different bulls; LDB, Lower Sperm Traits Profile among different bulls.

To evaluate the effect of isolated traits, due to correlations between all sperm traits analyzed, we could not select samples with extreme values in the ranks since values of higher and lower groups would also differ for the other traits. Therefore, although isolated effect of each sperm trait differed significantly between high and low groups, values of medians were not so distant. Values of median, upper and lower quartiles of groups formed are shown in supporting information S1 Table. It is noteworthy that values from all groups used in this study, even for the lower groups, were considerably elevated (MA, MI and MP > 70%, AI > 87% and CR > 96,9%). Nevertheless, we used only commercially available semen straws from reproduction centers, which have high quality criteria to release batches for commercialization. In addition, we used Percoll^®^ selection that improves sample quality. These are the same conditions of commercial IVP laboratories and therefore, ours results may well typify the current state of IVP.

### Step 3: Sperm traits effect on IVP yield

**Fig 2:**
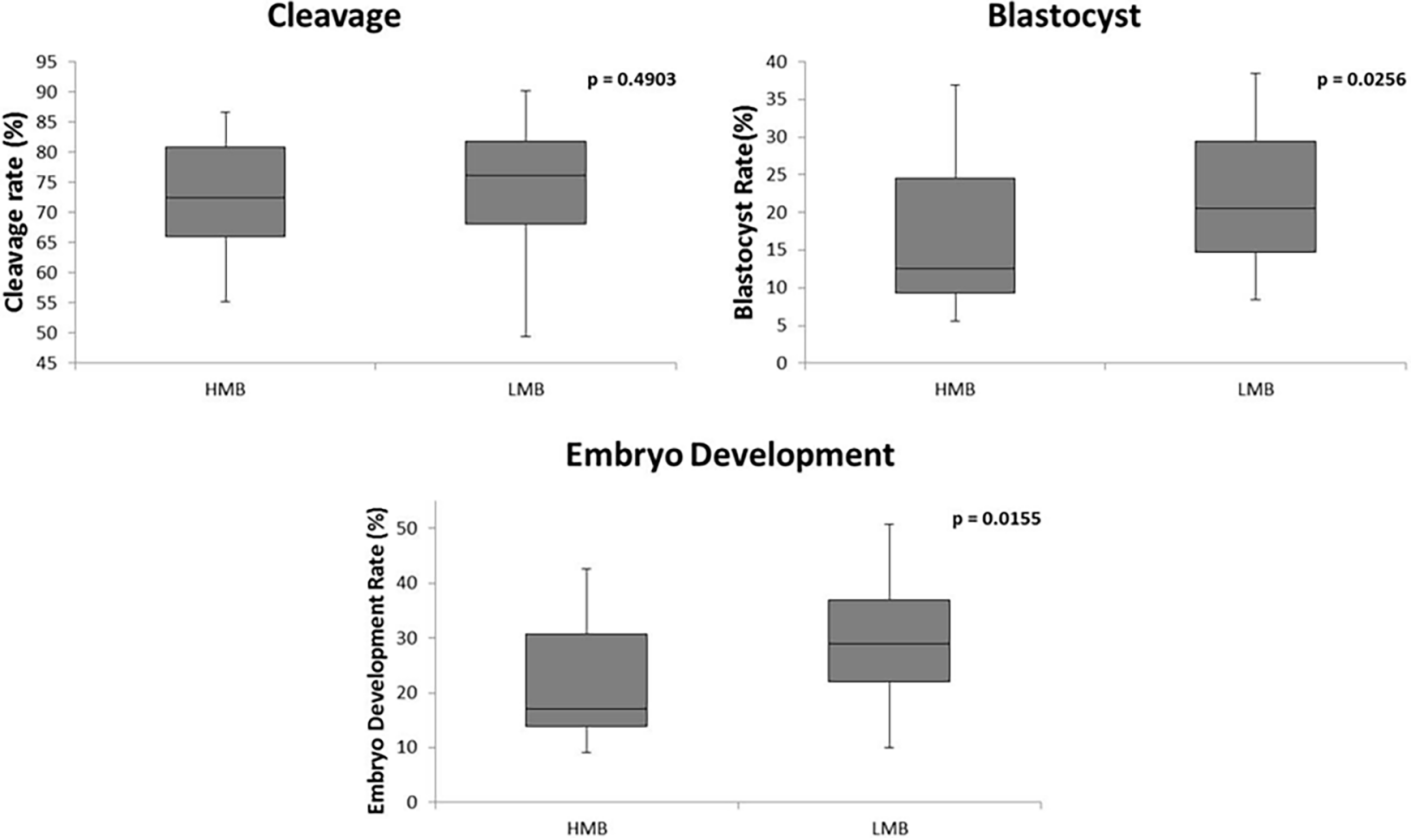
Comparison of cleavage, blastocyst, and embryo development rates between higher (HMB; n = 20) and lower (LMB; n = 20) motility before Percoll^®^. HMB, higher motility before Percoll^®^; LMB, lower motility before Percoll^®^.

**Fig 3:**
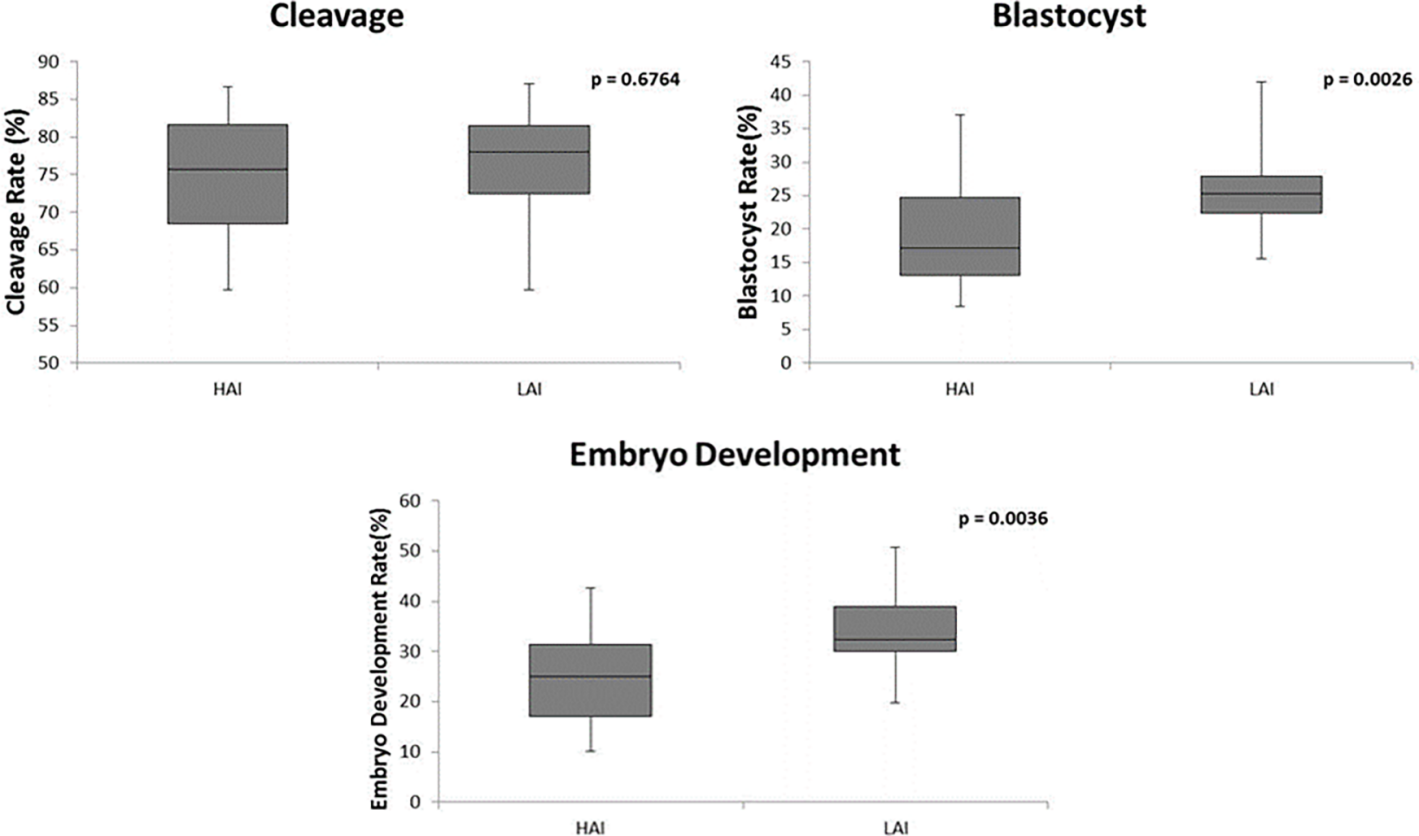
Comparison of cleavage, blastocyst, and embryo development rates between higher (HAI; n = 21) and lower (LAI; n = 20) acrosome integrity. HAI, higher acrosome integrity; LAI, lower acrosome integrity.

**Fig 4:**
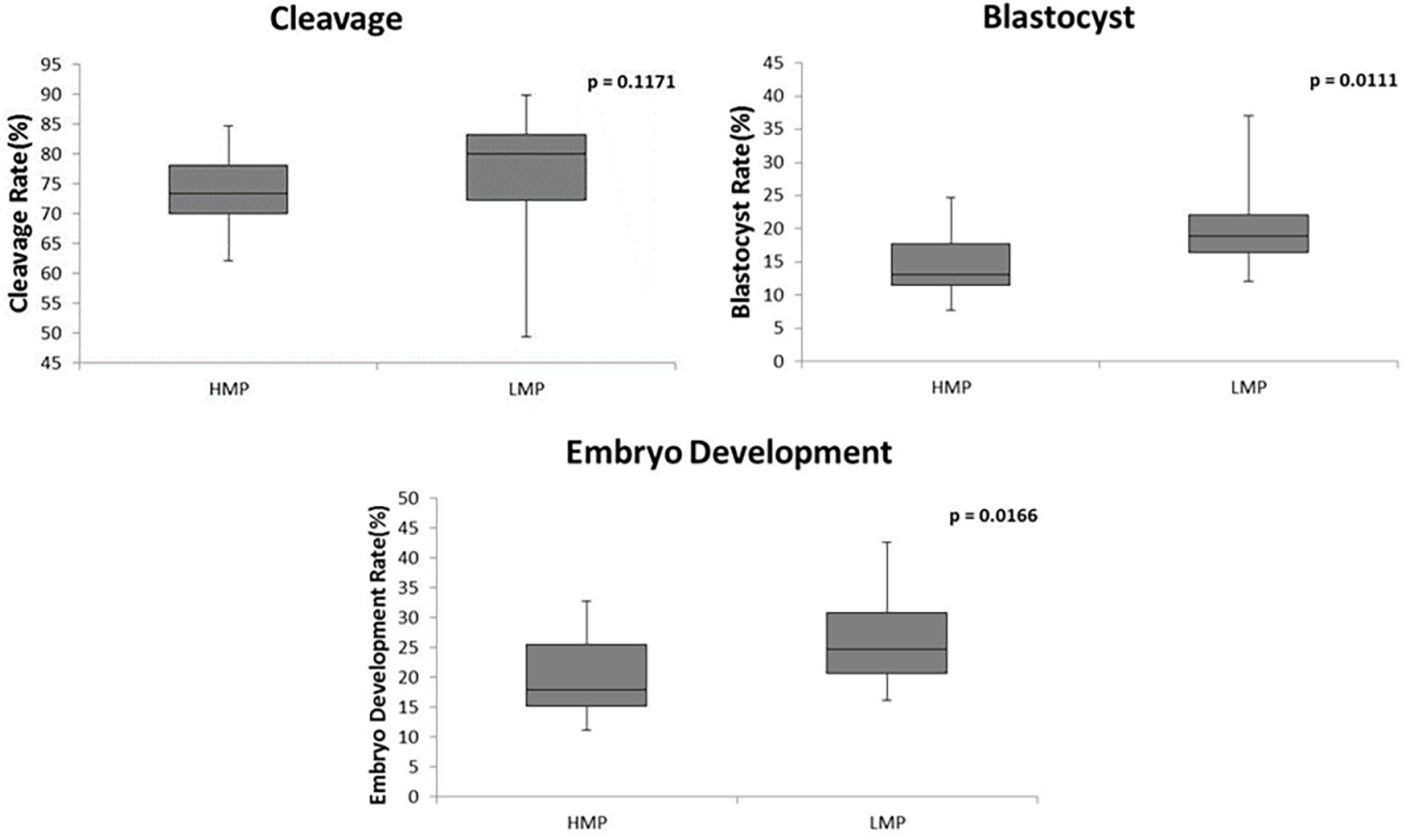
Comparison of cleavage, blastocyst, and embryo development rates between higher (HMP; n = 17) and lower (LMP; n = 16) mitochondrial membrane potential. HMP, higher mitochondrial membrane potential; LMP, lower mitochondrial membrane potential.

There was no difference for cleavage, blastocyst and embryo development rates when we evaluated the isolated effect of motility after Percoll^®^, membrane integrity, chromatin resistance, and the combined effect of all traits (Table 5). However, samples with lower motility before Percoll^®^ (Fig 2), or acrosome integrity (Fig 3) or mitochondrial membrane potential (Fig 4) resulted in higher embryo development and blastocyst rates (p<0,05), although there was no difference for cleavage rate. The IVP rates and p value of comparison among higher and lower groups of isolated effects and combined effects are shown in supporting information S2 Table.

**Table 5:** Comparison of IVP yields between groups. Comparison of cleavage, blastocyst and embryo development between higher groups and lower groups of isolated and combined effects.

Effect Studied	IVP YIELDS		
	Cleavage	Blastocyst	Embryo Development
Isolated Effect of Motility Before Percoll^®^ (HMB X LMB)	○	●	●
Isolated Effect of Motility After Percoll^®^ (HMA X LMA)	○	○	○
Isolated Effect of Acrosome Integrity (HAI X LAI)	○	●	●
Isolated Effect of Membrane Integrity (HMI X LMI)	○	○	○
Isolated Effect of Mitochondrial Potential (HMP X LMP)	○	●	●
Isolated Effect of Chromatin Resistance (HCR X LCR)	○	○	○
Combined Effect among Same Bulls (HSB X LSB)	○	○	○
Combined Effect among Different Bulls (HDB X LDB)	○	○	○

●, significant difference between higher and lower groups (p <0.05); ○, absence of significant difference between higher and lower groups (p ≥0.05); HMB, Higher Motility Before Percoll^®^; LMB, Lower Motility Before Percoll^®^; HMA, Higher Motility After Percoll^®^; LMA, Lower Motility After Percoll^®^; HMI, Higher Membrane Integrity; LMI, Lower Membrane Integrity; HAI, Higher Acrosome Integrity; LAI, Lower Acrosome Integrity; HMP, Higher Mitochondrial Membrane Potential; LMP, Lower Mitochondrial Membrane Potential; HCR, Higher Chromatin Resistance; LCV, Lower Chromatin Resistance; HSB, Higher Sperm Traits Profile among same bulls; LSB, Lower Sperm Traits Profile among same bulls; HDB, Higher Sperm Traits Profile among different bulls; LDB, Lower Sperm Traits Profile among different bulls

These results clearly showed that some improved sperm traits, such as higher percentages of sperm showing intact acrosome, higher mitochondrial membrane potential and higher motile cells before Percoll^®^ had a negative impact on blastocyst rate under *in vitro* conditions. The negative effect was irrespective of cleavage rate, only affecting the subsequently embryo development and blastocyst rate, showing a late effect of some sperm traits profiles used on IVF.

#### Effect of motility before Percoll^®^ (MB) on IVP rates

The higher blastocyst rate and embryo development of lower motility before Percoll^®^ (LMB) group was unexpected. Alomar et al. [14] assessed sperm quality parameters of six bulls relating the production of embryos with different abilities of *in vitro* development. These authors accidentally observed that two bulls from their study with the lowest motility before Percoll^®^ separation had the highest blastocyst rates, notwithstanding the significance of that observation was not tested. However, our study is the first to show consistently that, samples with lower motility before Percoll^®^ selection had a higher IVP performance. A possible cause of a lower total post-thaw motility could be suboptimal conditions during the cryopreservation process. On the other hand, the sperm subpopulation more resistant to the cryopreservation challenge is suggested to be more resilient to *in vitro* incubation in capacitating condition, to the osmotic stress, and also showing a more rapid and progressive pattern of motility [34,35]. In addition, this subpopulation shows higher longevity after thaw, indicating that those cells are the most probable source of fertilizing sperm [34,35]. Taking these facts into account, we suggested that LMB samples could be derived from a suboptimal cryopreservation condition resulting in lower number of motile spermatozoa. However, those cells that survived such selection pressure are probably the more resistant and, therefore, more prone to fertilize the oocyte. On the other hand, ideal cryopreservation conditions may allow the survival of increased number of sperm (higher post-thaw motility group) which, nevertheless, may not be necessarily the most competent. However, sperm motility patterns such as velocity and progression, as well as longevity during *in vitro* capacitating condition of these groups should be evaluated to confirm this hypothesis.

#### Effect of acrosome integrity (AI) on IVP rates

Analyses of sperm traits to predict fertility of bulls showed that acrosome integrity explained part of the variation on *in vitro* fertilization rates [13]. Acrosome defects and function alterations result in low *in vitro* fertility mainly due to reduced ability of spermatozoa to bind, penetrate, and decompact the DNA [7,8, 36,37]. Furthermore, oocytes penetrated by these spermatozoa had reduced potential of cleavage and embryo development [7], suggesting that acrosome function goes beyond the binding/penetration event. According to this, spermatozoa devoid of acrosome from males with globozoospermia failed to induce oocyte activation even after intracytoplasmic sperm injection (ICSI) [38]. However, other authors found no significant relation between acrosome status and *in vitro* fertility [9,14,39]. Besides the integrity of acrosome, the moment and amount of acrosome reaction have been shown to influence *in vitro* fertility, whereas absence, low and premature acrosome reaction reduce *in vitro* fertilization ability [37, 40–42].

In our study, the group of samples with lower acrosome integrity (LAI) resulted in higher embryo development and blastocyst rates than the group with higher acrosome integrity (HAI). Previous studies demonstrated that bulls with lowest *in vitro* fertility had a higher decrease of acrosome integrity during *in vitro* incubation [9,14]. Alomar et al. [14] suggested that acrosome content released into the IVF medium during this incubation could be detrimental to gametes. Corroborating with this hypothesis, Zambrano et al. [43] have recently demonstrated that the chemically removal of the acrosome improves embryo development and blastocyst rate on ICSI, indicating a negative effect of the hydrolytic enzymes from acrosome content on bovine oocytes. A higher amount of acrosome content released by HAI group creating a detrimental environment could explain the lower embryo development.

#### Effect of mitochondrial membrane potential (MP) on IVP rates

In our study, group with lower mitochondrial membrane potential provided higher embryo development and blastocyst rates. Chromosomal aberrations of bovine blastocysts caused by reactive oxygen species (ROS) generated from sperm with high mitochondrial membrane potential were already demonstrated [44]. Embryo development alterations as consequence of sperm oxidative stress have been clearly shown by studies in bull [44–46], rhesus macaque [47] and mouse [48]. Similarly, antioxidant treatment of sperm reduced ROS production and lipid peroxidation, while increased sperm quality and *in vitro* fertilization ability [49], and further protected spermatozoa from ROS inducer without affect *in vitro* development of embryos [50]. The fact that mitochondrial ROS generation is probably the most important source of ROS in sperm [51] and that sperm with high mitochondrial membrane potential exhibits greater potential to release pro-oxidative agents [46], endorse our hypothesis. We suggest that the negative effect of a higher mitochondrial membrane potential on blastocyst development is due to higher ROS generation in IVF environment resulting in oxidative damage of gametes and early zygotes. On the other hand, mitochondrial membrane potential has been associated to semen quality and fertility potential [21,25–27,30,52]. It is important to note that, *in vitro* conditions may be a stressing factor that should be considered when interpreting the present results. Higher mitochondrial potential may be an important trait when assessing a given semen sample quality; however, based on our results and on *in vitro* conditions, higher potential could be potentially deleterious.

#### The weak influence of motility after Percoll^®^ (MA), membrane integrity (MI) and chromatin resistance (CR) on IVP rates

Due to the slight differences of values between higher and lower groups, we cannot exclude the influence of these sperm traits on IVP yields. Here, we can only infer that IVP rates under actual conditions are more influenced by the motility before Percoll^®^, acrosome integrity and mitochondrial membrane potential of samples used in IVF step than the motility after Percoll^®^, membrane integrity and chromatin resistance.

#### Effect of combined sperm traits on IVP rates

Although combined effect groups showed the greatest differences of traits profile, we did not observe any differences on IVP rates. In addition, these sperm profiles had lower IVP yields than sperm profiles of isolated effect. We suggest that the combination of traits previously seen as detrimental in our study (higher mitochondrial membrane potential, higher motility before Percoll^®^ and higher acrosome integrity) could have an additive effect, decreasing drastically the IVP yields. On the other side, the association of lower levels of all sperm traits could impair the fertilization potential of these samples.

#### The late effect of IVF sperm profiles on blastocyst rates

In the present study, there was no effect for any of the traits tested on cleavage rates. We suggest that the effects of sperm traits are not involved with the fertilization ability but in fact they are involved on the environment generated during IVF by sperm samples with different traits profiles. Oxidative stress on gametes has a clear potential to cause a reduction of embryo development and blastocyst rates in several species [44–48]. This reduction could occur in the absence of cleavage impairment [44,45,47,48].

Kato and Nagao [44] showed that induced capacitation had a negative effect due ROS generation by mitochondria from motile capacitated sperm on embryo development, relating motility, acrosome status and mitochondrial membrane potential to ROS generation simultaneously. In addition, capacitation induced spermatozoa generates up to five times higher ROS than non-induced spermatozoa [44,53,54] and a previous sperm exposure to oxidative conditions enhances the ability of spermatozoa to generate ROS after capacitation induction [53]. In that way, the higher percentage of spermatozoa with acrosome integrity, which not underwent capacitation process, would endure the capacitation induced by IVF medium, generating higher amounts of ROS in the *in vitro* environment.

Concerning the motility before Percoll^®^, the higher percentage of motile sperm before Percoll^®^ could generates increased amounts of ROS in these samples, and this exposure to ROS previously to capacitation induction would enhance the ability of sperm from HMB group to generate ROS during IVF step. In light of such hypothesis, it is possible to postulate a role between ROS generation potential of sperm traits profile and IVP yields. Although we cannot determine the real pathways for the effects of some sperm traits on embryo development and IVP yields in the present study, we expect that further studies can elucidate such mechanisms.

In summary, sperm traits influence the *in vitro* embryo development. Higher motility before Percoll^®^, higher percentages of intact acrosome and high mitochondrial membrane potential could potentially decrease IVP yields, being among the main traits that define IVP yields of sperm samples.

## Supporting information

S1 Table Comparison of sperm profile between groups (Step 2). **Percentages (median, lower quartile, upper quartile) of sperm motility before Percoll^®^ (MB), motility after Percoll^®^ (MA), acrosome integrity (AI), membrane integrity (MI), mitochondrial membrane potential (MP) and chromatin resistance (CR) of higher groups and lower groups.** ^a,b^Values in the same column with different superscripts differ significantly; n, number of straws analyzed; HMB, Higher Motility Before Percoll^®^; LMB, Lower Motility Before Percoll^®^; HMA, Higher Motility After Percoll^®^; LMA, Lower Motility After Percoll^®^; HMI, Higher Membrane Integrity; LMI, Lower Membrane Integrity; HAI, Higher Acrosome Integrity; LAI, Lower Acrosome Integrity; HMP, Higher Mitochondrial Membrane Potential; LMP, Lower Mitochondrial Membrane Potential; HCR, Higher Chromatin Resistance; LCV, Lower Chromatin Resistance; HSB, Higher Sperm Traits Profile among same bulls; LSB, Lower Sperm Traits Profile among same bulls; HDB, Higher Sperm Traits Profile among different bulls; LDB, Lower Sperm Traits Profile among different bulls.
(DOCX)

S2 Table Comparison of IVP yields between groups (Step 3). **Cleavage, Blastocyst, Embryo Development Rates (median-%) and P-Value to comparison between higher groups and lower groups of isolated and combined effects.** ^a,b^Values in the same column with different superscripts differ significantly; n, number of IVP procedures considered; P, p-value.
(DOCX)
