# Supplementary material for: Sperm traits on in vitro production (IVP) of bovine embryos: Too much of anything is good for nothing
Source: PLoS One. 2018 Jul 10;13(7):e0200273. doi: 10.1371/journal.pone.0200273 (PMC6039049; doi:10.1371/journal.pone.0200273)
Supplement: S1 Table — Percentages (median, lower quartile, upper quartile) of sperm motility before Percoll® (MB), motility after Percoll® (MA), acrosome integrity (AI), membrane integrity (MI), mitochondrial membrane potential (MP) and chromatin resistance (CR) of higher groups and lower groups. a,bValues in the same column with different superscripts differ significantly; n, number of straws analyzed; HMB, Higher Motility Before Percoll®; LMB, Lower Motility Before Percoll®; HMA, Higher Motility After Percoll®; LMA, Lower Motility After Percoll®; HMI, Higher Membrane Integrity; LMI, Lower Membrane Integrity; HAI, Higher Acrosome Integrity; LAI, Lower Acrosome Integrity; HMP, Higher Mitochondrial Membrane Potential; LMP, Lower Mitochondrial Membrane Potential; HCR, Higher Chromatin Resistance; LCV, Lower Chromatin Resistance; HSB, Higher Sperm Traits Profile among same bulls; LSB, Lower Sperm Traits Profile among same bulls; HDB, Higher Sperm Traits Profile among different bulls; LDB, Lower Sperm Traits Profile among different bulls. (DOCX) [file pone.0200273.s001.docx]

| Isolated Effect of Motility Before Percoll^®^ | | | |
| --- | --- | --- | --- |
| Trait | **HMB (n=20)** | **LMB (n=20)** | **P value** |
| MB (%) | **60.0 (60.0; 67.5)^a^** | **40.0 (32.5; 50.0)^b^** | **<0.0001** |
| MA (%) | 75.0 (70.0; 80.0) | 72.5 (67.5; 75.0) | 0.2187 |
| MI (%) | 80.8 (75.4; 84.2) | 79.7 (76.1; 83.3) | 0.4326 |
| AI (%) | 90.8 (88.3; 92.8) | 92.2 (90.9; 94.6) | 0.1134 |
| MP (%) | 87.8 (83.6; 89.2) | 86.6 (80.5; 89.1) | 0.5272 |
| CR (%) | 98.9 (98.1; 99.4) | 98.4 (96.5; 99.5) | 0.4013 |
| Isolated Effect of Motility After Percoll^®^ | | | |
| Trait | **HMA (n=20)** | **LMA (n=20)** | **P value** |
| MB (%) | 60.0 (45.0; 60.0) | 55.0 (47.5; 67.5) | 0.7415 |
| MA (%) | **80.0 (72.5; 82.5)^a^** | **70.0 (60.0; 70.0)^b^** | **0.0006** |
| MI (%) | 78.5 (73.1; 82.3) | 78.2 (75.3; 84.2) | 0.6167 |
| AI (%) | 90.8 (88.5; 94.3) | 88.7 (87.8; 92.7) | 0.2791 |
| MP (%) | 85.6 (83.0; 88.2) | 83.2 (79.9; 88.1) | 0.4488 |
| CR (%) | 98.4 (97.9; 99.2) | 98.6 (97.1; 99.1) | 0.5423 |
| Isolated Effect of Membrane Integrity | | | |
| Trait | **HMI (n=20)** | **LMI (n=20)** | **P value** |
| MB (%) | 60.0 (45.0; 60.0) | 60.0 (45.0; 60.0) | 0.9680 |
| MA (%) | 72.5 (70.0; 80.0) | 70.0 (70.0; 80.0) | 0.9776 |
| MI (%) | **81.0 (78.2; 83.4)^a^** | **75.6 (72.2; 78.6)^b^** | **0.0012** |
| AI (%) | 89.1 (87.6; 91.7) | 88.7 (87.8; 91.7) | 0.9568 |
| MP (%) | 86.3 (83.5; 88.6) | 85.1 (81.2; 87.5) | 0.2856 |
| CR (%) | 98.7 (97.4; 99.3) | 98.7 (97.7; 99.1) | 0.8284 |
| Isolated Effect of Acrosome Integrity | | | |
| Trait | **HAI (n=20)** | **LAI (n=20)** | **P value** |
| MB (%) | 42.5 (30.0; 60.0) | 52.5 (40.0; 62.5) | 0.2677 |
| MA (%) | 70.0 (65.0; 80.0) | 75.0 (70.0; 80.0) | 0.4083 |
| MI (%) | 77.8 (72.6; 81.9) | 78.6 (73.1; 80.7) | 0.8923 |
| AI (%) | **91.95 (89.9; 93.9)^a^** | **88.7 (87.9; 89.9)^b^** | **0.0033** |
| MP (%) | 86.6 (83.9; 87.7) | 84.9 (80.8; 88.0) | 0.4327 |
| CR (%) | 98.3 (96.8; 99.4) | 98.0 (97.0; 98.4) | 0.2553 |
| Isolated Effect of Mitochondrial Membrane Potential | | | |
| Trait | **HMP (n=16)** | **LMP (n=16)** | **P value** |
| MB (%) | 47.5 (32.5; 60.0) | 50. (40.0; 60.0) | 0.5942 |
| MA (%) | 70.0 (70.0; 72.5) | 70.0 (70.0; 80.0) | 0.2470 |
| MI (%) | 77.3 (69.9; 79.1) | 73.7 (68.5; 78.3) | 0.3270 |
| AI (%) | 89.3 (86.8; 91.9) | 87.9 (85.5; 90.8) | 0.3270 |
| MP (%) | **86.6 (84.4; 88.8)^a^** | **75.9 (72.8; 81.8)^b^** | **0.0029** |
| CR (%) | 98.2 (96.6; 99.3) | 98.3 (96.1; 98.8) | 0.4284 |
| Isolated Effect of Chromatin Resistance | | | |
| Trait | **HCR (n=20)** | **LCR (n=20)** | **P value** |
| MB (%) | 45.0 (40.0; 55.0) | 45.0 (30.0; 60.0) | 0.7435 |
| MA (%) | 70.0 (67.5; 75.0) | 70.0 (65.0; 72.5) | 0.4541 |
| MI (%) | 79.9 (75.9; 84.3) | 78.3 (73.4; 80.5) | 0.1555 |
| AI (%) | 91.5 (88.1; 93.7) | 90.7 (87.9; 92.2) | 0.4568 |
| MP (%) | 86.7 (83.6; 90.3) | 86.3 (83.1; 88.7) | 0.3039 |
| CR (%) | **99.0 (98.7; 99.4)^a^** | **96.9 (96.4; 98.6)^b^** | **0.0004** |
| Combined Effect among Same Bulls | | | |
| Trait | **HSB (n=16)** | **LSB (n=16)** | **P value** |
| MB (%) | **60.0 (42.5; 62.5)^a^** | **40.0 (30.0; 50.0)^b^** | **0.0081** |
| MA (%) | **70.0 (67.5; 75.0)^a^** | **60.0 (50.0; 70.0)^b^** | **0.0294** |
| MI (%) | **79.8 (75.2; 84.1)^a^** | **67.5 (53.9; 74.5)^b^** | **0.0006** |
| AI (%) | **91.0 (87.2; 94.3)^a^** | **80.3 (71.4 88.3)^b^** | **0.0006** |
| MP (%) | **86.8 (84.8; 88.7)^a^** | **74.6 (66.4; 82.7)^b^** | **<0.0001** |
| CR (%) | **98.7 (96.5; 99.1)^a^** | **97.7 (89.0; 98.5)^b^** | **0.0345** |
| Combined Effect among Different Bulls | | | |
| Trait | **HDB (n=16)** | **LDB (n=16)** | **P value** |
| MB (%) | **60.0 (50.0; 65.0)^a^** | **32.5 (30.0; 55.0)^b^** | **0.0059** |
| MA (%) | **75.0 (70.0; 82.5)^a^** | **62.5 (52.5; 70.0)^b^** | **0.0064** |
| MI (%) | **86.5 (84.6; 88.2)^a^** | **60.1 (53.9; 65.5)^b^** | **<0.0001** |
| AI (%) | **95.7 (94.2; 96.5)^a^** | **76.0 (71.1; 82.1)^b^** | **<0.0001** |
| MP (%) | **88.9 (87.9; 91.0)^a^** | **68.7 (64.2; 77.5)^b^** | **<0.0001** |
| CR (%) | **99.3 (99.1; 99.7)^a^** | **95.3 (86.6; 98.1)^b^** | **0.0002** |
