# Supplementary material for: Sperm traits on in vitro production (IVP) of bovine embryos: Too much of anything is good for nothing
Source: PLoS One. 2018 Jul 10;13(7):e0200273. doi: 10.1371/journal.pone.0200273 (PMC6039049; doi:10.1371/journal.pone.0200273)
Supplement: S2 Table — Cleavage, Blastocyst, Embryo Development Rates (median-%) and P-Value to comparison between higher groups and lower groups of isolated and combined effects. a,bValues in the same column with different superscripts differ significantly; n, number of IVP procedures considered; P, p-value. (DOCX) [file pone.0200273.s002.docx]

| Cleavage | | | | | |
| --- | --- | --- | --- | --- | --- |
| Effect | **Higher** | **N** | **Lower** | **n** | **P** |
| Isolated - Motility Before Percoll^®^ | 72.4 | 20 | 76.1 | 20 | 0.4903 |
| Isolated - Motility After Percoll^®^ | 79.2 | 20 | 74.1 | 18 | 0.6191 |
| Isolated - Acrosome Integrity | 75.7 | 21 | 78.0 | 20 | 0.6764 |
| Isolated - Membrane Integrity | 74.4 | 20 | 76.7 | 20 | 0.2732 |
| Isolated - Mitochondrial Potential | 73.4 | 17 | 80.0 | 16 | 0.1171 |
| Isolated - Chromatin Resistance | 77.4 | 19 | 78.4 | 21 | 0.8709 |
| Combined - Same Bulls | 70.4 | 17 | 77.2 | 15 | 0.3647 |
| Combined - Different Bulls | 70.4 | 17 | 77.1 | 16 | 0.1548 |
| Blastocyst | | | | | |
| Effect | **Higher** | **N** | **Lower** | **n** | **P** |
| Isolated - Motility Before Percoll^®^ | **12.5^a^** | **20** | **20.5^b^** | **20** | **0.0256** |
| Isolated - Motility After Percoll^®^ | 22.9 | 20 | 17.9 | 18 | 0.8493 |
| Isolated - Acrosome Integrity | **17.1^a^** | **21** | **25.3^b^** | **20** | **0.0026** |
| Isolated - Membrane Integrity | 17.6 | 20 | 20.5 | 20 | 0.2977 |
| Isolated - Mitochondrial Potential | **13.1^a^** | **17** | **18.9^b^** | **16** | **0.0111** |
| Isolated - Chromatin Resistance | 19.7 | 19 | 20.2 | 21 | 0.8496 |
| Combined - Same Bulls | 10.1 | 17 | 9.8 | 15 | 0.5583 |
| Combined - Different Bulls | 9.8 | 17 | 16 | 16 | 0.1445 |
| Embryo Development | | | | | |
| Effect | **Higher** | **N** | **Lower** | **n** | **P** |
| Isolated - Motility Before Percoll^®^ | **17.1^a^** | **20** | **28.9^b^** | **20** | **0.0155** |
| Isolated - Motility After Percoll^®^ | 29.6 | 20 | 23.3 | 18 | 0.8037 |
| Isolated - Acrosome Integrity | **24.9^a^** | **21** | **32.2^b^** | **20** | **0.0036** |
| Isolated - Membrane Integrity | 24.5 | 20 | 24.1 | 20 | 0.3369 |
| Isolated - Mitochondrial Potential | **18.0^a^** | **17** | **34.7^b^** | **16** | **0.0166** |
| Isolated - Chromatin Resistance | 25.8 | 19 | 25.8 | 21 | 0.7452 |
| Combined - Same Bulls | 16.6 | 17 | 14.6 | 15 | 0.5970 |
| Combined - Different Bulls | 15.9 | 17 | 21.2 | 16 | 0.1655 |
